# The Viking viewer for connectomics: scalable multi-user annotation and summarization of large volume data sets

**DOI:** 10.1111/j.1365-2818.2010.03402.x

**Published:** 2011-01

**Authors:** JR ANDERSON, S MOHAMMED, B GRIMM, BW JONES, P KOSHEVOY, T TASDIZEN, R WHITAKER, RE MARC

**Affiliations:** * Department of Ophthalmology, Moran Eye Center, University of UtahSalt Lake City, Utah, U.S.A.; † Scientific Computing and Imaging Institute, University of UtahSalt Lake City, Utah, U.S.A.; ‡ Sorenson MediaSalt Lake City, Utah, U.S.A.; § Department Electrical and Computer Engineering, University of UtahSalt Lake City, Utah, U.S.A.

**Keywords:** Annotation, automated electron microscopy, citizen science, computational methods, connectome, networks, visualization

## Abstract

Modern microscope automation permits the collection of vast amounts of continuous anatomical imagery in both two and three dimensions. These large data sets present significant challenges for data storage, access, viewing, annotation and analysis. The cost and overhead of collecting and storing the data can be extremely high. Large data sets quickly exceed an individual's capability for timely analysis and present challenges in efficiently applying transforms, if needed. Finally annotated anatomical data sets can represent a significant investment of resources and should be easily accessible to the scientific community. The Viking application was our solution created to view and annotate a 16.5 TB ultrastructural retinal connectome volume and we demonstrate its utility in reconstructing neural networks for a distinctive retinal amacrine cell class. Viking has several key features. (1) It works over the internet using HTTP and supports many concurrent users limited only by hardware. (2) It supports a multi-user, collaborative annotation strategy. (3) It cleanly demarcates viewing and analysis from data collection and hosting. (4) It is capable of applying transformations in real-time. (5) It has an easily extensible user interface, allowing addition of specialized modules without rewriting the viewer.

## Introduction

Automated microscopy systems, in combination with automated registration algorithms, are allowing microscopists to collect data sets of unprecedented scale. The utility of this ability is typified by descriptive studies of neural connectivity, which is the assembly of neural connectomes. Neural features span six to nine orders of magnitude. Axons can extend from 10 to 10^6^ μm; dendritic arbors can subtend less than 10 μm or over 1000 μm; single cells may make one or thousands of connections and interact with 1–12 different classes of target cells; individual synapses and gap junctions subtend 0.1–1 μm, and anatomical pairings can be validated only with resolutions of 2 nm or better. Our lab focuses on retinal networks and our first small neural volume (RC1) required capturing large amounts of serial section transmission electron microscopy data (Anderson *et al.*, 2009): about 341 000 images, at 16 megapixels each. Browsing and annotating the myriad features of such massive data sets, as well as summarizing attributes and relationships in human-understandable forms, are substantial computational undertakings. We present our solution, *Viking*, in the context of our retinal connectivity work tracing the AII amacrine cell of the retina.

The development of Viking began shortly before the capture of the RC1 data set and it was ready for use upon completion of capture. Viking addressed several core problems. After capturing the raw data we used the NCR Toolset (publicly available at http://sci.utah.edu/software.html) to calculate transformations for positioning tiles into canonical images, and for aligning images as a volume. The NCR Toolset did not include a viewer. The NCR Toolset does export assembled, transformed images, but the immense size of a single canonical image (10–20 GB downsampled to 8-bits) is too large for 32-bit image formats, incompatible with commercial tools, and doubles the storage footprint. Even for 32-bit compliant images the export process is time-consuming, as each pixel must be mapped. Furthermore, when producing images aligned into the volume, every pixel must be cascaded through each intermediate slice-to-slice (stos) transform, and this process becomes progressively slower as the number of transforms (size of the volume) increases. Beyond the need to view full-resolution images, we needed to rapidly monitor and debug data acquisition or transformation errors. At the time we began acquisition of RC1, the NCR Toolset had been tested on smaller volumes, but it was not known whether the tools would scale to a volume of over 340 000 tiles. We needed a data viewer which would accept any transform and apply it to the original data in real-time using graphical processing unit speeds.

The second problem was the need for a network-oriented client–server solution. When volume collection began, the largest consumer hard drive could store 1 terabyte (TB). RC1 occupied 16.5 TB after processing so the volume had to be stored on a central file server and shared. As more connectomes are acquired, at higher speeds, the ‘enterprise’ scale of connectomics data will outstrip the growth in commercial desktop hard drive storage in the near term. Our single TEM is now capable of generating 0.25 TB of processed images per day and acquisition can be multiplexed across microscopes. The obvious solution was to expose all images and transformations via HTTP and have the viewer load the requested data on demand.

The third problem was annotation. Our tasks included identifying target cells, tracing processes through the volume, recording instances of synapses or gap junctions, tagging novel features emergent in browsing the data and tagging each cell with the molecular identities acquired from intercalated optical imaging (Anderson *et al.*, 2009). We use the term ‘tracing’ to describe this activity, and we consider a cell traced when all of its processes and features have been annotated. Cells are complex in texture and topology and, as yet, there is no fully-automated markup strategy for such imagery. Even if such tools existed, they would still require human validation (Mishchenko, 2009). There is no escaping the need for fast, robust, shared annotation tools. A rough estimate of the time required to annotate RC1 shows that, even if a single human could locate and mark each feature on a conventional computer display at an inhumanly fast rate of ten per minute, annotating an estimated 10^7^ structures (e.g. every subsegment of every process) would require at least 5.7 work years. Conversely, the use of network-compliant viewers and annotation means that small teams of analysts can easily trim work times by 10-fold. Another accelerating feature of our particular approach to connectomics we incorporated into our viewer is the use of computational molecular phenotyping (Marc & Jones, 2002). By using molecular signals to classify cells, we may not have to trace every cell in a volume, but rather only a representative set from each cell class. The annotation system also required flexibility. Our focus on circuitry should not limit the annotation systems applicability to other problem areas as these issues are not unique to neural cells. Both serial section transmission electron microscopy and viewers like Viking can be powerful adjuncts to the study of complex heterocellular tissues in general.

As analysts mark up a data set, they should be able to benefit from each other's work. For example, discovering annotations of a nearby interacting cell provides context for probable markup sites in a cell of interest. For users who wish to construct models, connectivity data should reside in a single database that also provides a venue to cross-validate annotations. Any annotation effort on this scale quickly produces at least 10^5^–10^6^ annotations that need to be summarized in a human-understandable format. Use of SQL database designs provided a powerful query language as the foundation for more sophisticated visualization and reporting tools. We concluded the scale of connectomics data mandated a multi-user, collaborative annotation strategy with the data stored in an enterprise-scale SQL database.

**Fig. 1:**
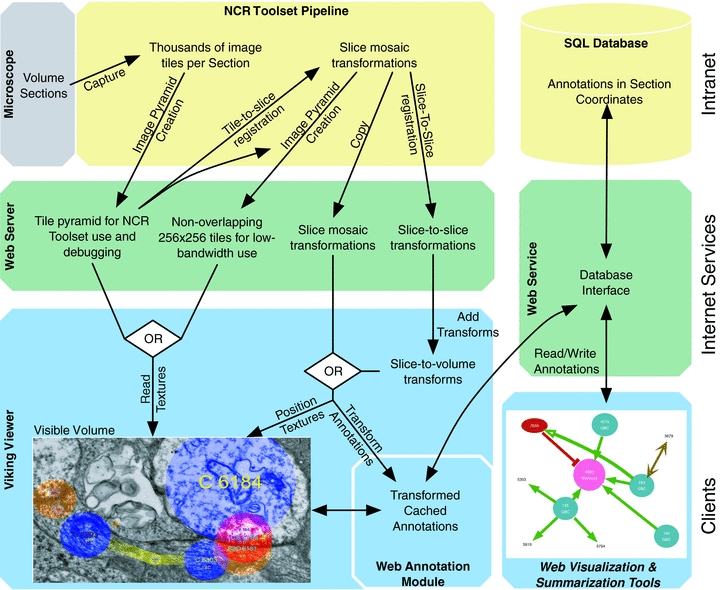
Overview of NCR Toolset and Viking systems. Viking provides a scalable environment for concurrent annotation, based on a three-tier architecture. Top-tier processing of the original data and database maintenance are performed on-site and need not be placed on the internet. Images, transforms, and a web services definition language (WSDL) interface form the middle tier to which a variety of client applications can be targeted. Client software includes our viewing/annotation client and visualization web site. Method calls between layers are stateless. Tiered designs allow modification of a layer without changes to the others, so long as the interfaces unchanged. Modular elements can be re-engineered if a new component is needed.

Viking is our solution to these problems. It uses a multi-tiered architecture consisting of viewer and analysis clients using HTTP to communicate with an image server and a WSDL compliant web server which in turn populates a backend SQL database (Fig. 1). It provides several key features. (1) It works over the internet using HTTP and supports many concurrent users limited only by hardware. (2) It supports a multi-user, collaborative annotation strategy. (3) It cleanly demarcates viewing and analysis from data collection and hosting. (4) It is capable of applying transformations in real-time. (5) It has an easily extensible user interface, allowing addition of specialized modules without rewriting the viewer.

## Materials and methods

### Language

Unless otherwise noted, software was written in Microsoft C# using Visual Studio 2008. SQL Server Express 2008 was used to store annotations. XNA 3.0 was used for image rendering. The Windows Communications Foundation was used for the web service. Matlab 2009b was used to visualize cell morphology. Python 2.6 scripts were used to invoke the NCR Toolset.

### Data

Our first retinal connectome volume (RC1) is a 33 μm tall, 0.25-mm-diameter image cylinder. It consists of 371 individual sections, of which 341 were captured as ≈1000 transmission electron microscope (TEM) images each at a resolution of 2.18 nm per pixel, plus 27 scaled optical images tagged for molecule markers. Eight images cap the column at each end and 11 are evenly spaced, 30 sections apart, within. We refer to the acquisition process as automated TEM (ATEM). RC1 occupied 16.5 TB of storage after processing.

The area and volume to be captured in any study are scaled by the complexity of the neural elements involved. In principal, each region of brain has a canonical element that contains copies of all cell classes required for its fundamental information processing operations (Anderson *et al.*, 2009). Similarly, canonical functional elements can be defined for other continuous domain tissues (liver, kidney). The canonical element serves as a reference for the completeness of any sample. In neural systems, the size of a canonical element varies with brain region and may not even be known for some areas. In serial section transmission electron microscopy, the volume element is cut into thin slices (≈70 nm in RC1), each containing a canonical area subtending the canonical element. We refer to that area as a canonical image and it is built from hundreds to thousands of image tiles. When we refer to a slice below, we really mean the canonical area for that slice, because the physical tissue section is much larger than the captured canonical area. Reassembling the volume from tiles and slices is a major undertaking. Our automatic solutions involve a series of algorithms implemented in the NCR Toolset (publicly available at http://sci.utah.edu/software.html). The NCR Toolset generates two transformations for each slice. The mosaic transformation describes where tiles fit inside a slice. The stos transformation describes how to register the entire slice mosaic to an adjacent slice.

### Volume description

Volumes are described by an XML file on the server. This file lists every slice in the volume, URLs for both mosaic and stos transformations (if present) and the URL where image data can be located. This allows a volume to be load-balanced across multiple systems, although we currently house the entire volume on a single system. The XML file also contains the URL for the web service used to annotate the data, allowing each volume to be annotated independently or to have multiple databases if needed. The XML definition is flexible enough to allow display of any tiled image data that follows a predictable naming convention. We have used Viking to view bright field, confocal, fluorescent and fundoscopic images.

### Transformations

**Fig. 2:**
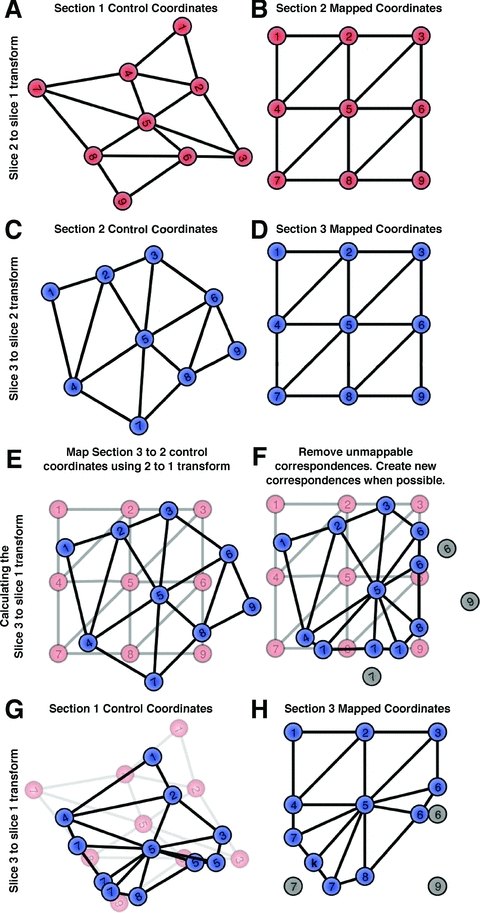
Transform addition. To create a volume for three slices, we begin with two stos transforms mapping slice 2 to slice 1 (2 → 1) (A,B) and slice 3 to slice 2 (3 → 2) (C,D). Initial transforms are constructed by placing an even grid of mapped points on the slice to be transformed (B,D). Control points are located on the control slice by the NCR Toolset (A,C). Viking loads the transforms and creates a slice-to-volume transform by mapping control points on slice 2 from the 3 → 2 transform onto the 2 → 1 transform (E). In some cases, control points cannot not be mapped because they fall outside the defined grid transform. When this occurs, each line connecting the point to its neighbours, determined by Delaunay triangulation, is tested to see if it intersects with the edge of the grid transform (F). The mapped coordinates are moved along the corresponding line by the same relative distance (H). The mapped control points create a new set of control points which define the 3 → 1 transform (G), which would be the slice-to-volume transform for slice 3 (G,H). After a small number of slices are transformed into a volume the mapped coordinates converge to a circular shape.

The first task Viking faces is creating a slice-to-volume transformation for each slice from the available stos transforms. How these transforms are generated is described briefly in Anderson *et al.* (2009) and in detail in Tasdizen *et al.* (in review). Each stos transform defines a grid of control points evenly spaced across the warped slice with a corresponding grid of points positioned on the stationary control slice. Viking designates a slice as the reference slice or origin for the volume and generates transforms required to register all other slices onto it (Fig. 2).

Currently, the lowest numbered slice in the series typically serves as the volume origin but, in theory, specifying an origin in the centre of the volume could reduce accumulated warps from transformation errors. The current volume is derived from 371 physical slices, of which 341 were able to be digitized. The minimal distortion produced by registering our entire volume to slice 1 (Anderson *et al.*, 2009) suggests that volumes of at least 700 slices are easily possible.

Different levels of refinement are available for volume registration. When using the NCR Toolset, multiple stos registration passes are made using 32× (32-fold), 16× and 8× downscaled mosaics as increasingly high-quality input. Each pass builds upon the previous registration. When traversing each 2-fold scaling, we maintain the spacing of grid points, resulting in a 4-fold increase in the number of registration points with each increase in resolution. This allows larger coarse adjustments followed by fine local refinements. Typically, we find the 16× registration produces high enough quality that we rarely use the 8× data due to slower startup times.

### The viewer

**Fig. 3:**
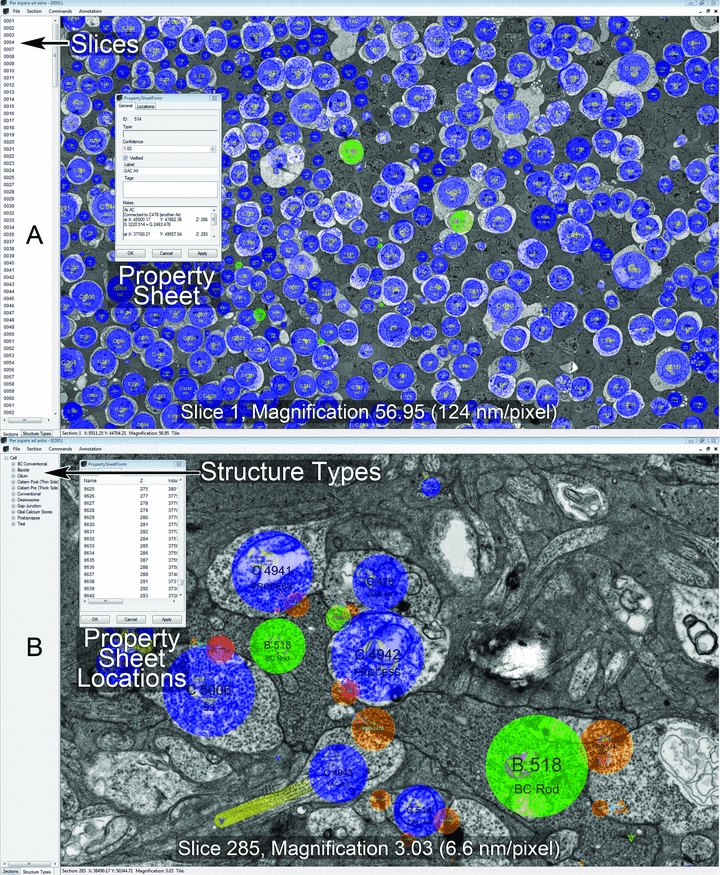
The Viking interface modes. (A) The Slice mode, selected at left tab window, allows selection of the slice to view in the right data window. Panning, zooming, and navigating forward or back a slice are mouse actions. Navigation is aided by keystroke shortcuts. This is a low-resolution (124 nm per pixel) view of an array of annotated cells (blue and green circles) in the inner nuclear layer of rabbit retinal connectome volume RC1. Individual structures (e.g. cells) all have Property Sheets for metadata logging and user guidance. (B) The Structure Type mode enables the annotation process. This is a high-resolution (6.6 nm per pixel) view of annotated processes and synapses. Figure 5 describes the annotation process. Property Sheets also have dynamically updated Location panels to guide navigation along a structure in X pixel, Y pixel Z slice space.

**Fig. 4:**
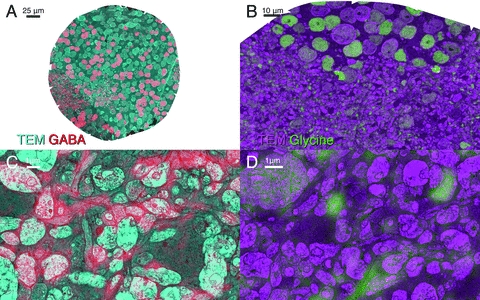
Multi-resolution and multi-channel support. (A,B) Low-magnification views of slice 61 (A) which contains a GABA overlay (red) captured with a 100× oil objective overlayed on the adjacent TEM section (cyan) and slice 152 (B) which contains a Glycine (green) overlayed on TEM (magenta). Note cell bodies containing GABA/Glycine are easily identified. (C,D) High-resolution views of from slice 182 w/GABA (C) and slice 152 with a Glycine overlay (D). Many fine processes can be identified as containing glycine or GABA. Brighter light microscopy overlays indicate higher concentrations of the target small molecule.

**Fig. 5:**
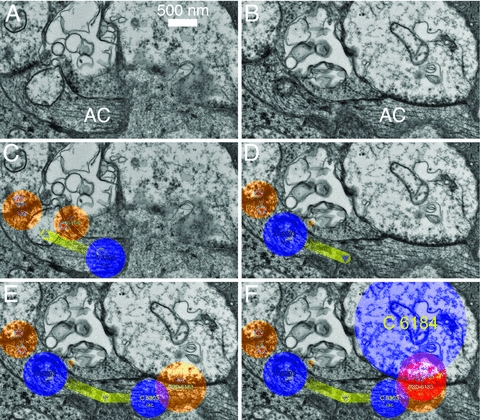
Tracking workflow in Viking. (A,B) Viking screen captures show unmarked EM images of two adjacent serial slices numbered 152 (A) and 153 (B). A long amacrine cell (AC) process passes horizontally along the bottom of both images. It is broken into two isolated segments on slice 153. (C) Annotation of slice 152 shows the AC process as structure C5303 (small blue circle) with two associated postsynaptic densities where input is received from unlabeled conventional synapses. Triangles indicate the locations of annotations on adjacent slices. Yellow lines indicate links between locations. (D–F) Progressive annotations of slice 153. (D) The left-hand segment of amacrine cell is annotated C5303, but the right-hand segment is not. (E) The user extends the tracking of C5303 by dragging a line from the existing location on the adjacent slice (triangle) which creates a new location (right-hand blue circle), a link line (yellow) and an entry in the LocationLinks table linking them. The user then creates a structure for the post-synaptic density (orange circle) and associates it with C5303 (fine white link line). (F) At a later time, a user tracks cell C6184 and finds that it forms the presynaptic part of the synapse annotated in (E). A conventional synapse structure (red circle) is created for the presynaptic side and linked to both C6184 and the paired postsynaptic structure with a drag–drop operation.

Viking allows viewing of a single slice at a time (Figs 3–5). The highly anisotropic voxels produced by ATEM, and the lack of need for a true volume rendering to extract synaptic networks, led us to the simple solution of displaying one slice of the volume at a time, and flipping through them like pages in a book. Viking performs real-time transformation of the original tiles using the fast texture mapping abilities of graphical processing units. This involves mapping each tile into slice space and then mapping into the volume space. We use the transform's mapped points placed evenly on the source tile grid as the u,v texture coordinates and map those points into either the slice or volume space to determine the tile's position. A cached Delaunay triangulation generates the required triangle mesh. As a result Viking can quickly switch between different transforms generated from any stage in the pipeline, because this only involves swapping out the vertices used for rendering. This real-time transformation ability strongly distinguishes Viking from previous viewers.

Tile images are loaded directly into texture memory as 8-bit greyscale images and are downloaded over HTTP as needed. Viking calculates the visible area and the downscale level for the camera's current window position, then determines which tiles are visible and the resolution required to display the data. Tiles not already in memory are loaded from a local disk cache, if available. Otherwise an HTTP request is made for the correct tile from the web server. This is a common strategy used in internet-enabled viewers. (Mikula *et al.*, 2007; Wernecke, 2008; Google, 2010)

Optimal performance of the NCR Toolset requires the original overlapping image tiles to be downscaled by factors of two and stored in an image pyramid. Viking is capable of displaying the volume using these tiles to verify the output of the tools. However, this is not an efficient structure for network viewing because the size of the images varies with the degree of downscaling, and the tiles have a 15% overlap, requiring download of redundant information. At full resolution a single screen could require downloading four 16 MB tiles and the lowest resolution requires downloading a thousand 64 × 64 pixel tiles, each making its own HTTP request. To resolve this problem Viking supports a second image pyramid configuration where all tiles at all levels of the pyramid have a fixed size (currently 256 × 256 pixels) and do not overlap. There is, thus, an upper bound placed on the number of tiles required to cover the screen and the small tile size allows rapid loading of textures over the network. Assembly of the optimized tiles from raw data is, itself, an unoptimized process requiring about 3 h of processing time for a 16 GB slice on an eight-core 3Ghz Mac Pro. However, the process need only be run once and further slice-to-volume transformations are done in real-time.

### Multi-channel and multi-resolution image support

Viking supports multi-channel and multi-resolution data by allowing any number of image pyramids to be associated with a slice and not requiring that image pyramids have the same number of levels. Users may overlay any number of sections and channels, assigning each a colour which are currently blended using simple addition of RGB values.

This feature is important for tracing because it allows us to incorporate non-TEM slices into TEM volumes. During the construction of RC1, sections capping and interleaved through the volume were collected onto glass slides and probed with antibodies against small molecules: 4-aminobutyrate (GABA), glycine, glutamate, glutamine, 4-amino-guanidobutane (agmatine), aspartate and taurine. Signals were detected with secondary antibodies conjugated to 1.4 nm gold particles and visualized using silver intensification (Marc & Jones, 2002). Images were captured on a Leica DMR microscope with Surveyor montaging software (Objective Imaging, Cambridge, U.K.), oversampled at 73 nm per pixel resolution. The optical images were aligned into mosaics using the NCR Toolset and manually registered (ir-tweak, NCR Toolset) to the nearest adjacent TEM slice, after the TEM slice was downsampled by a factor of 32 (70 nm per pixel). The registered image was exported and divided into a 256×256 image pyramid and included in the volume as an additional channel for the TEM slice with maximum available resolution of 70 nm per pixel. In future volumes, optical microscopy slices will be assigned their own slice number (a design oversight in RC1) and use their own slice-to-slice transformation so the original images are warped into the volume. The volume description file optionally defines the default visible channels on a per-section or per-volume level. The RC1 configuration overlays EM images with the small molecule imagery when such data is available allowing the users to identify whether a cell they are tracing contains a given small molecule (Fig. 4).

### The Module system

The drawback to placing the transformation mathematics in the viewer is the difficulty of creating viewers for other specialized tasks. To address this, Viking uses an extension module system built upon NET reflection. At startup, Viking checks a directory for DLL files and examines them for types exposing specific attributes and interfaces. Any objects found are added to the user interface. If no modules are found Viking functions only as a viewer. The entire annotation system described later is included in Viking as an optional module. The user workflow for annotations is described in Fig. 5.

### The annotation server

The annotation database is stored on a Microsoft SQLExpress 2008 server and exposed via HTTP using a Windows Communication Foundation web service on Internet Information Server. This service exposes a number of methods to read and write annotations invokable via simple object access protocol. For example, when Viking loads a slice it invokes a method on the web service that returns either all annotations on the slice and adjacent slices, or all annotations that have changed if the slice was visited previously. The web service returns plain text XML strings with objects formatted in Java Script Object Notation for compatibility with different software clients. This meets future needs of sharing databases (Amari *et al.*, 2002).

### The annotation database

**Fig. 6:**
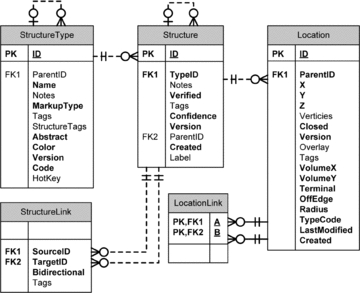
The Viking annotation database SQL schema. Each box contains the table name (top panel), a list of columns (right panel), and whether the column serves as a key in the database (left panel). The functions of these tables are described in the Annotation Database portion of the methods. Primary keys are noted with PK, Foreign keys are denoted with FK. Column names listed in bold are required fields. Relations (lines) between tables are shown using Crow's foot notation.

The annotation schema was designed to be flexible and not restricted to neural network markups. This allows users to survey and choose from existing rich ontologies (Martone *et al.*, 2008) or define their own. We accomplished this by allowing users to place annotations in both graph and tree data structures. Significant tables contain XML ‘Tag’ columns to provide a structured mechanism for extending entities with user-defined attributes. Figure 6 shows a high-level overview of the database schema. Column names are indicated in italics text with parenthesis.

#### *The structure type table* .

This table describes the types (classes) of biological structures that will be tracked through the volume. It exposes an optional parent field (*ParentID*) so relationships can be enforced. For instance, Cell is a top-level structure type in RC1. We also have a Presynaptic Ribbon Synapse structure type, but it is a child of the cell type (cell.ribbon). This informs the Viking client that ribbon annotations can only exist in association with a parent cell. The Viking client provides an interface to edit this table so users can define their own ontology.

#### *The structure table* .

This table describes instances of each structure type. Each structure is assigned a unique identifier (*ID*). Each structure has a type (*TypeID*). The type determines if the structure is required to point to a parent structure (*ParentID*) if that relationship is specified in the StructureType tree. Thus, an instance of our ribbon synapse type would have a unique ID, and the ParentID field would specify which cell it belonged to. As a structure may span many slices in the volume, we do not store geometry or position information in this table.

#### *The location table* .

The location table describes where structures appear on specific slices. Tracing a cell ID through the volume involves populating this table with the all locations for that cell ID. All locations are stored in slice space (*X*,*Y*,*Z*), not volume space. This prevents future refinements of the slice-to-volume transformation from corrupting existing annotations in the database. As Viking loads the locations for each slice, they are transformed to the volume space. However the last valid Volume position is cached in the database (*VolumeX*, *VolumeY*) to simplify the creation of visualization tools that should not be required to implement the transformations. Locations can be currently described as points or circles (*Radius*, *TypeCode*). Masks and polygons are planned enhancements (*Vertices*, *Closed*), although they have performance implications in a multi-user environment. When users track a process off the edge of the volume or reach a dead end flags are set to indicate this (*OffEdge*, *Terminal*).

#### *The location links table* .

This table stores adjacency information for cell ID locations (*A*,*B*) so that graphs of the physical layout and connectedness of any structure can be constructed. This table is essential, as cells have processes which frequently bifurcate and make dozens of different appearances on a single slice. This also allows us to model which location belongs to which process, and permits queries such as travel distance between two locations. It is also used for three-dimensional renderings of annotated structures. At a finer scale, synaptic connections are extended structures, not merely points, and subtend characteristic areas and volumes. This is an important parameter, as synaptic strength can be classified by the volume of presynaptic vesicles or area of postsynaptic specialization (Kasai *et al.*, 2003; Sheng & Hoogenraad, 2007).

#### *The structure links table* .

This table stores structure connections regardless of physical location, and the resulting graph describes these relationships. Each link has a source (*SourceID*) and a target (*TargetID*). Links can be directional. In our schema, cells are not connected in this table directly. Rather, they contain child structures which may be connected. We defined several connective structure types for cells: presynaptic (conventional, ribbon), postsynaptic densities and gap junctions (nominally bidirectional for markup). Each connection is built from two half-connections, typically identified separately at markup time. This is only the schema convention we have adopted. Users are capable of defining their own ontologies that may or may not restrict which structures can be linked. A natural extension of this concept to heterocellular tissues in general would include child structures such as zonulae occludens or adherens, desmosomes, endothelial fenestrations, and extracellular matrix domains.

### Analysis

As the database is populated with morphology and connectivity information it becomes essential that we transform the data to human understandable formats. Morphology visualization is Matlab based, while graph visualizations are hosted on a web page which retrieves annotations via the web service. This approach conveniently partitions analysis tools from the data, promotes the wide dissemination of results, and allows third parties to perform their own analysis without storing the data.

### Morphology

**Fig. 7:**
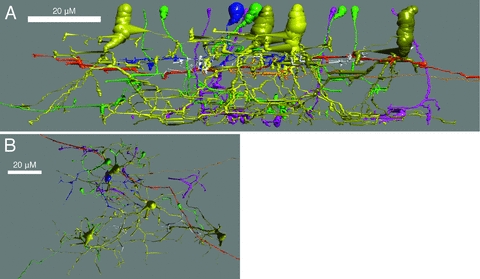
Rendering of cells in VikingPlot. Selected neurons connected in an A_II_ amacrine cell network were rendered with the VikingPlot, demonstrating their approximate morphologies in vertical (A) and horizontal (B) orientations. The A_II_ amacrine cells are numbered 476, 514, 2610 and 3679 in Fig. 8, which shows the connectivity graph. Cell classes are colour coded. A_II_ amacrine cells are shades of yellow. ON Bipolar cells are shades of green. OFF BCs are shades of blue. Rod bipolar cells are shades of purple. Unidentified processes are white. A long OFF α ganglion cell dendrite (C5150) entering from the right in (A) is coded red-orange. A process containing peptide vesicles (C6406) also enters from the right (A) slightly below and is colour light orange.

The Location and LocationLinks tables encode graphs of the physical locations of structures in the volume from which morphology can be visualized (Fig. 7). We used Matlab 2009b (http://www.mathworks.com), Matlab Database Toolbox, and the Matlab Compiler to create a command-line tool, VikingPlot, which is passed a set of StructureIDs at startup. VikingPlot then queries the database for the locations of each structure and any child structures. Each location is converted in a circle of vertices. Polygon faces are created using Matlab's ‘patch’ command to connected locations as defined by the LocationLinks table. This creates a cylinder between each connected location. If a location has fewer than two links, a sphere is rendered to cap the cylinder. We use this tool to visualize morphology as we annotate the database in near real-time, displaying large networks of cells and examining the stratification of synapses and gap junctions in different cell classes. However, this tool is again applicable to any complex cell shape. A web-based morphology rendering tool is in development. The graph visualization web page also supports displaying cells as stick diagrams. Process branch points and terminations serve as nodes and the Euclidean distance between them is calculated to determine edge length. This allows questions of synaptic spacing or cable properties to be addressed.

**Fig. 8:**
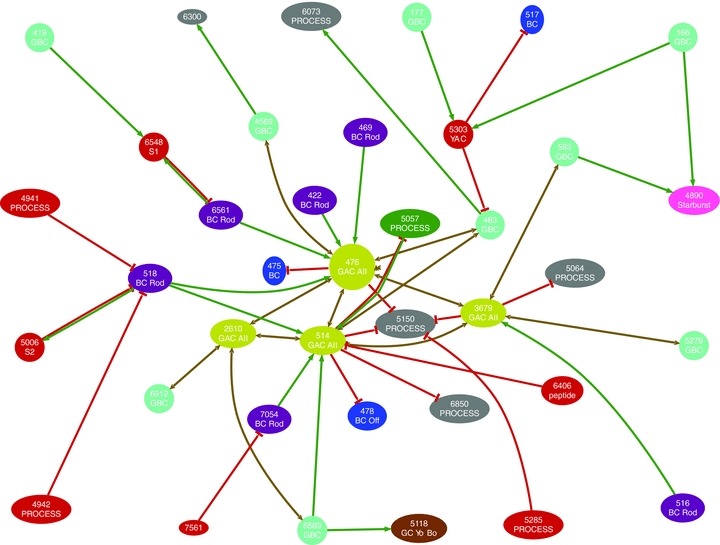
Automatic generation of network graphs. The visualization web query (see ‘Materials and methods’) for cells connected to A_II_ amacrine cell 476 by three or fewer hops produces a directed graph with an automated ‘pretty’ layout for human interpretation. Nodes are colour coded according to cell type or are grey if type has not been assigned. Solitary numbers indicate that the graph continues past the requested hop limit. Edges are directed: green arrows represent ribbon synapses, red T-bars represent conventional synapses, yellow double headed arrows represent gap junctions. Labels: BC, bipolar cell; GC, ganglion cell; AC, amacrine cell; GBC, glycine immunopositive bipolar cell; PROCESS, element not yet classified.

### Networks

The penultimate product of a connectome project is a network diagram, formally represented as a cyclic, directed multigraph with cells as vertices and synapses/gap junctions as arcs (edges). We automate generation of our network diagrams from the annotation database using the Structures and StructureLinks tables (Fig. 8). The visualization web page allows the user to request the connectivity graph for a structure. A graph is then constructed containing all edges and structures within a user-defined number of hops from the requested structure. The layout is generated by GraphViz (Gansner & North, 1999). A scalable vector graphics file is generated for web-viewing or editing. Users can hover over any edge in the graph to retrieve a list of structures responsible for the edge. This enables quick verification or image export in the Viking viewer. For our project, this is one kind of summary display that represents terabytes of raw data as a lightweight, publishable figure. Once again, these tools can be generalized for other tissues by generating adjacency graphs or network graphs of adhesion patterns.

## Results

We have used the Viking system to map substantial portions of the scotopic network of the mammalian retina (Kolb & Famiglietti, 1975; Strettoi *et al.*, 1990; Strettoi *et al.*, 1992) by tracing five neighbouring A_II_ amacrine cells; related rod bipolar, coupled ON cone bipolar and OFF cone bipolar cells; target OFF centre ganglion cells; related amacrine cells and their inputs, cascading to a range of connected cells (Figs 3, 5, 7, 8). The RC1 annotation database currently stores over 200 000 locations and over 8000 structures (including cells, synapses, gap junctions, etc.). While Viking was created to view and annotate RC1, it is currently used as the primary viewer for 10–30 GB images produced as the routine output of a general high-throughput TEM facility. This has been a very productive, successful environment for cell biological and neuroscience research and discovery. In the process, user feedback motivated design changes. Since data viewers tend to converge on similar design patterns we will share the rationale and outcomes of our decisions.

### Real-time transformation

Making the viewer perform real-time image transformation may seem an unnecessary burden, but provides several advantages. The alternative would be to write the warped image files in advance (a nontrivial task) and have the viewer read the finished images. Although this would lead to faster performance when navigating a finished volume, real-time transformation allows us to preview the volume while it is being constructed. For example, occasionally slices are placed on a grid upside down, so the resulting images have to be flipped. Sometimes the reported order of the slices is incorrect and this is discovered only after annotation. Occasionally, prospective improvements to algorithms in the NCR Toolset to improve the registration quality need to be tested, and we thus regenerate the stos transformations. Slices with occasional tears, folds or other defects may make them unsuitable for pairwise stos alignment and we need to substitute alternate, defect-free slices as pairwise references. As stos transforms are cascaded to create the slice-to-volume transforms, correcting the registration of one slice in mid-volume would mean regenerating all images downstream of the repair. Without real-time transformation, the entire volume would have to be reconstructed *ab initio*, taking 6 weeks using optimized multi-threaded CPU-based code on our current build machine (8-core 3Ghz Mac Pro). Real-time transformation addresses all of these problems by allowing us to examine the transformations generated by each stage of the pipeline. Viking allows us to identify, isolate and correct the causes of errors. Further, extensions to a volume in the form of re-imaging at higher resolution, different grid tilts or mapping outside the original capture field can best be accommodated with real-time transformation. Using real-time warping means that any changes are automatically instantiated by Viking on start-up.

A second major advantage to having the transformations available to the viewer is flexibility in annotation. We store all locations in slice space, not volume space. This allows us to change or improve volume transformation without breaking existing annotations. It also allows us to display annotations correctly even if the slice is not transformed into the volume. The database stores the last calculated volume position to facilitate data export to other applications.

### Speed

The speed at which users can annotate the volume is critical. Key factors include the time to load images into memory over a network and the physical manipulation of controls to create annotations. Viking has been a work-in-progress and the speed with which users can view the volume is constantly improving. We have gradually incorporated a number of common optimizations, such as asynchronous loading of annotations, only loading annotations which have changed since the last visit, and caching of transformations and tiles. At this time a user can fully trace one complex cell per day and several simple ones (e.g. rod bipolar cells).

Our approach to network tracing also requires us to address the ergonomics of annotation. By initially limiting ourselves to annotations using circles, we allowed the users to extend structures with only two mouse clicks. We use a drag-and-drop convention for extending, linking and resizing annotations. Although circles produce rather coarse visualizations, they create a good approximation of structure dimensions for modelling (indeed likely better than most models can accommodate) and do not hinder network discovery in any way. As we began our tracing efforts it also became clear that users needed to be able to navigate the volume quickly. Viking provides a mechanism to jump to any X,Y,Z coordinate using copy and paste keys, or by entering coordinates into a location dialog. We are also able to search within Viking for any location of interest on a structure.

Easy navigation facilitates tracing cells. Typically, users open a property dialogue for the structure they are tracing and, as a complicated structure is traversed (such as a dendritic arbor), they record the coordinates of branch nodes and follow a path. When the first path is exhausted, they jump back to the node and follow a second path. When a branch terminates, the user sets a flag. Users can also use the property page for a structure to see a list of all locations for a structure and double-click any location to jump to it. This is useful when trying to locate a specific structure in a slice with hundreds of annotations. Terminal locations that have not been explicitly flagged are searchable, providing a mechanism to detect unmapped or overlooked processes.

### The collaborative annotation database

**Fig. 9:**
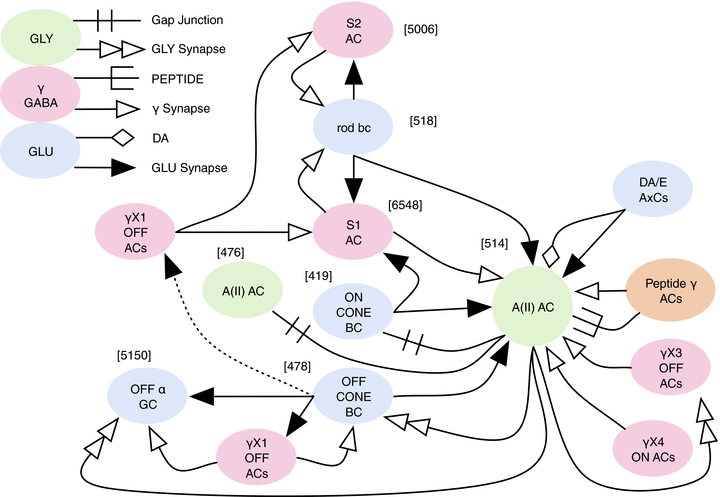
Network summary. Using automated network graphs, connectivity data were further condensed to present the connectivity of multiple A_II_ amacrine cells as a single human-interpretable summary. Combining CMP, morphological and ATEM connectivity observations makes it possible to classify cells in the observed network by multiple techniques in the same data set.

The annotation database is becoming increasingly useful as a resource for exploring new research questions. Beyond basic queries for the connectedness or non-connectedness of cells, the graph-like layout of the annotations (Fig. 8) make it an excellent output for modelling applications or generating concrete summaries (Fig. 9). Other queries may address contacts or structures not directly involved in signalling and information processing, but whose heterocellular patterning may be clues to developmental rules, such as desmosomes, or homeostatic roles, such as intracellular organelles. Viking's markup schema is flexible and allows us to create new child structures which become important biological markers. For example, the non-neural Müller cells of the retina contain a novel organelle (a packet of organized smooth endoplasmic reticulum) and its spatial distribution and dimensions within the Müller cell became a critical question. Viking serves as a general tool of discovery and annotation for histomics: the analysis of ultrastructural relationships within any tissue.

The collaborative annotation database is an essential resource of validated (e.g. human-validated) data for training of automated tracing systems. As an example, it was trivial to query the database for over 1000 examples of ribbon synapses it contains. We passed those coordinates to the batch image export function of Viking to create a library of validated training images for an automatic feature recognition project.

### HTTP-based collaboration

Viking's multi-tiered architecture scales well with additional hardware and care was taken during implementation to support a large number of users. Methods invoked across tiers are stateless, allowing each request to be handled independently and irrespective of order which facilities load distribution. Although we have not had the means to perform large scalability tests over the last year, we have routinely had three users annotating simultaneously and have tested with six workstations concurrently running the Viking client and accessing volume RC1 without any user noticing performance limitations. This includes concurrent use by local and international annotators. The multi-user approach is key for accelerating discovery with manual tracing. It is not uncommon for one investigator to find a connection to a cell previously traced by a collaborator. We can also re-trace cells for validation. Tracing a new process through a densely annotated region is much faster, as it eliminates candidates for a process as it traverses a tangle of fine structures in a slice transition or it identifies incipient connection sites.

The use of a web service allows the decoupling of data and clients. Both our viewer and visualization web page require only HTTP and do not require any privileged status such as a direct database connection to function. The advantages are that any institution can query our annotations and images without having to replicate the data. Database replication needs to be avoided in a collaborative annotation system because any copies quickly diverge. By using a standard interface we can also point our visualization tools at the web services of collaborators using the Viking system with no installation requirement. As a result, it is easier to share data and tools between groups as long as interface definitions are maintained (Amari *et al.*, 2002).

## Discussion

Viking is an extensible tool for connectomics analysis and is generalizable to histomics applications. We sought to produce a practical viewing and annotation implementation using existing technologies, so we could begin data mining immediately. The Viking system is not the first and will not be the last software written for anatomical data. Brainmaps.org provides an excellent internet viewer for mesoscale anatomical data including annotation support (Mikula *et al.*, 2007). Although Viking was developed independently, these tools are nevertheless examples of convergent development, in which similar designs emerged for sharing large-scale image data over the internet. Reconstruct is a richer environment for single-user tracing of individual structures at the ultrastructural scale (Fiala, 2005). Viking's strengths are real-time transformation of images, a user-defined annotation ontology, high annotation throughput and public interfaces which allow anyone to create clients to read images or edit annotations. Creation of a web service client is automated in many programming environments, such as Matlab and Visual Studio.

We chose to use circles as structural elements in annotation. We avoided the use of user-defined image masks, as this would have been too slow (at least by a factor of 10) for mapping networks from both the ergonomic and computational perspectives. We were not willing to slow annotation, because our focus was networks. Structure rendering, of course, is a different objective. There are also engineering challenges in implementing masks for a multi-user environment. For example, associating a full-resolution bit-mask with each location in the database at 1:1000 compression would require a 2 MB download to retrieve a fully masked slice if the viewer was zoomed out. With good heuristics it would be possible to retrieve a subset, but additional time is required to decompress and manage textures. Even so, we recognize image masks as an important tool which we are working towards. With the RC1 database in a state of rich annotation, masks will permit a new, more precise class of biological queries, such as classifying synaptic topologies or measuring organelle dimensions. We plan to allow user-guided automation, perhaps relying on the user to generate an initial masks, which is propagated to later slices and refit.

**Fig. 10:**
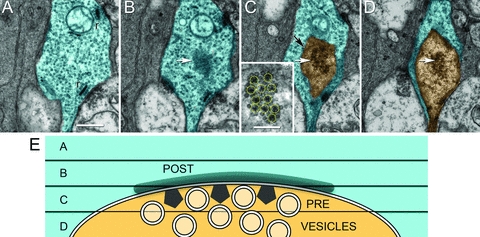
Serial slices demonstrating the challenges in automated tracking. Oblique orientation synapses are common. Four adjacent 70 nm slices in the RC1 volume illustrate the problem, which cannot be solved by making slices thinner. (A) In slice 232, a bipolar cell process (cyan) is centred in the frame, with two ribbon (r) synapses. There are actually four oblique synapses in this series. We track only one. Scale for (A–D), 500 nm. (B) On slice 231, a central dark patch represents the bipolar cell's postsynaptic density (white arrow) to an amacrine cell input. (C) In slice 230, the amacrine cell process (orange) emerges in the centre of the contact zone (white arrow) along with its array of presynaptic projection densities (inset box, yellow circles, scale, 250 nm). The membranes between the amacrine cell and bipolar cell pass through the slice obliquely and appear as a dark circular smudges (black arrow). Presynaptic vesicles appear in the centre of the patch. (D) Slice 229 contains the amacrine cell process (orange) with its cluster of presynaptic vesicles (white arrow) rimmed by the bipolar cell (cyan). (E) An orthogonal view of the four-slice partitioning of a conventional synapse with the bipolar cell (cyan) postsynaptic density (POST) in slice B, the amacrine cell (orange) presynaptic projections (PRE) in slice C, and vesicles (circles) in slice D.

With a robust user-guided annotation in place, we can now explore semi-automated annotation. Although a new grand challenge in connectomics is automated markup, generating the primary connectome data set was enough of a challenge itself that adding dependencies on automated tracing systems made no sense. Proof-reading semi-automated annotations can be nearly as slow as manual markup (Mishchenko, 2009). Greater automation of tracing appears possible and would be an important step in speeding analysis. However, subsets of the problem domain vary greatly in difficulty. Automated tracing of bipolar cell axons down to their target levels in the inner plexiform layer is fairly simple. Automated tracing of fine amacrine processes as they traverse laterally through the volume or labelling oblique synapse orientations (Fig. 10) are much harder tasks.

We have found that manual tracking of fine oblique features is rarely difficult. TEM images are projection images, where electrons pass through the entire slice imaging features at all depths. By contrast, back-scattered electron signals, such as those produced by block-face scanning electron and ion beam milling platforms, contain little depth information. For TEM, importantly, the challenge of automatic or manual identification of oblique synapses is not significantly improved using finer Z resolution obtained with thinner sections which lower contrast and spread the identification task across more slices. In critical cases we could, in principle, acquire a tomographic image series of a process in question to track an oblique process, but in practice we have never needed to do this.

**Fig. 11:**
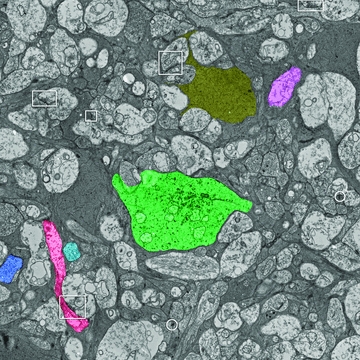
A keyframe image for the RC1 514_ipl tracking challenge. Six cells have been manually masked off and they are numbered in order of difficulty, with cell #1 being the easiest to track. The goal is to track the labelled cells from any starting section to the edges of the challenge volume. The challenge volume was exported from Viking at full resolution and starts at slice 126 (location X 48888, Y 48888) in the inner plexiform layer of the retina, centred on A_II_ amacrine cell C514 (green). Selected processes are colour coded to guide initiation of tracking (yellow, Müller cell; blue, bipolar cell 1; cyan, bipolar cell 2; red, amacrine cell; purple, ganglion cell). A selection of some (not all) representative conventional (boxes) and ribbon (circles) synapses is also provided. The image is 17.8 μm wide.

Viking's annotation database provides a wealth of expert knowledge for testing and annotation. The modular nature of the viewer also makes it a good platform for experimentation. Although acknowledging that are other ‘grand challenge’ automated tracing data sets exists, dense synaptic systems such as retina offer a maximally complex problem domain. Thus, we have used Viking to export a full resolution test series for tracking from the RC1 data set (Fig. 11). The tracking challenge data set 514_ipl has a resolution of 2.18 nm and is composed of ninety 8192 × 8192 64 MB subslices from slices 126–219. A 5 GB LZW compressed data set and key guide images are publicly available at http://prometheus.med.utah.edu/~marclab/connectome_tracking_2009.html.

We will release the Viking viewer and web visualization tools as a resource to access our connectome in the third quarter of 2010 (available at http://connectomes.utah.edu). By doing this we hope to decouple the collection and storage of data from the discovery and analysis components allowing the entire scientific community to benefit from connectome data sets. Similar to the proliferation of genome and transcriptome mining tools (e.g. Mungall *et al.*, 2002), we hope to promote a wide variety of online tools from many groups. We will also transfer the RC1 data set to user-supplied media on request.

Concurrent use of the annotation database brings up issues of how conflicting updates are resolved. This is a large topic and most of the functionality is implemented by underlying software libraries. We considered how to handle conflicting inserts, updates and delete actions. For the insert case we simply allowed the duplication. If two users trace the same cell they would notice the conflicting activity quickly because slice annotations are updated each time the user switches slices or presses the refresh key. If two users trace the same cell at the same rate loading slices at exactly the same time, it is possible to have a duplicate tracing. In reality one user will always be slightly faster and the slower user would see the annotations appear over the cell they were tracing and know they should delete their progress within a slice or two. Duplicate annotations are also easily identified automatically by searching for overlapping annotations. For the conflicting update case we took a common approach of including a version timestamp column on tables with update operations. If the row version has changed between the time a users performs the query and submits the update to a row a message is displayed and the changes are rejected. When masks are adopted we plan to adopt the convention that each pixel can only belong to a single structure and treat conflicts as conflicting updates. The final case is deletion. If user A deletes a location or structure which user B is attempting to update or link with an error message is displayed.

Including the small molecule imagery in the RC1 volume is a boon to both understanding both network organization and error checking annotations. Small molecules diffuse throughout intracellular space. They permit users to validate that the same molecular signature is present in the process they are tracing and that a mistake has not been made. This can also benefit automated tracking and error detection strategies by providing an independent method to measure correctness.

One of our long-term goals, shared with many other groups, is to relieve the analysis bottleneck of large data sets by enabling citizen science: massive community annotation of data volumes. Similar to space science efforts such as GalaxyZoo for analysis of Sloan Deep Sky Survey data (Schawinski *et al.*, 2009), and the NASA/JPL-Caltech/Microsoft project BeAMartian (NASA *et al.*, 2010) that hosts Mars image data for citizen markup, future progress in neuroscience (and perhaps bioscience in general) will depend on reifications driven by annotations. We acknowledge that perhaps in the end only neuroanatomists will be motivated to trace circuits, however the features to support a community of anatomists, such as user identity and change logging, are identical to the features to support a community of volunteers or paid workers. There are not and will never be enough analysts in all the neuroscience labs worldwide to carry out the critical annotations one might envision. The number possible of connection topologies in any brain region is so diverse that only measured ground truth will suffice (Anderson *et al.*, 2009) and the diversity of brain regions, genetic models, disease-related rewiring topologies and therapeutic models is overwhelming. This venture could easily engage exabyte to zettabyte data scales. The only practical way forward in these early days of connectomics research, even with semi-automated markup is to make data sets public and enable community markup. This would make it possible to enlist the aid of advocacy groups for diseases such as Alzheimer's and epilepsy, as well as a spectrum of cognitive disorders with developmental or environmental origins. Citizen science has several attractive features, new to hypothesis-driven science perhaps, but intrinsic to database-enabled systems like *Viking*. First, no participant in markup, analysis, reification or other use of the data has to physically own the database. This is a major economic issue and represents the ultimate form of data sharing. Secondly, overlapping annotations of the same data set opens the possibility for self-correction, especially when emergent models can be viewed by many specialists. In a similar way, scientific bias or even outright fraud becomes virtually impossible. This radically changes the way perhaps all forms of scientific investigation might be done in the future, with validation coming not from the slow process of peer-review but rather from rapid, anonymous exploration of data sets. One objection might be that only specialists are qualified to annotate databases. We have found that to be untrue. Undergraduates and other novice analysts with little or no neuroscience training can readily learn to perform annotation based on visual recognition alone, especially when validated by an experienced analyst early on. The very positive experience of the Galaxy Zoo effort to use citizen science engaging over 160 000 users to classify astronomical objects (Schawinski *et al.*, 2009) is consistent with ours. Furthermore, we have found that naïve analysts can discover features that experienced observers do not, perhaps due to bias or narrow focus.
